# Biosynthesis of magnetite and cobalt ferrite nanoparticles using extracts of “hairy” roots: preparation, characterization, estimation for environmental remediation and biological application†

**DOI:** 10.1039/d1ra04080d

**Published:** 2021-08-09

**Authors:** Natalia Kobylinska, Dmytro Klymchuk, Anatolij Shakhovsky, Olena Khainakova, Yakiv Ratushnyak, Volodymyr Duplij, Nadiia Matvieieva

**Affiliations:** A. V. Dumansky Institute of Colloid and Water Chemistry, NAS of Ukraine Ak. Vernadsky blv. 42 Kyiv 03142 Ukraine kobilinskaya@univ.kiev.ua; M. G. Kholodny Institute of Botany, NAS of Ukraine 2 Tereshchenkivska Str Kyiv 02000 Ukraine; Institute of Cell Biology and Genetic Engineering, NAS of Ukraine 148 Zabolotnogo Str. Kyiv 03143 Ukraine; University of Oviedo 8 Julián Claveria Av. Oviedo 33006 Spain

## Abstract

The “green” synthesis of magnetite and cobalt ferrite nanoparticles (Fe_3_O_4_-NPs and CoFe_2_O_4_-NPs) using extracts of *Artemisia annua* L “hairy” roots was proposed. In particular, the effect and role of important variables in the ‘green’ synthesis process, including the metal–salt ratio, various counter ions in the reaction mixture, concentration of total flavonoids and reducing power of the extract, were evaluated. The morphology and size distribution of the magnetic nanoparticles (MNPs) depended on the metal oxidation state and ratio of Fe(iii) : Fe(ii) in the initial reaction mixture. MNPs obtained from divalent metal salts in the reaction mixture were non-uniform in size with high aggregation level. Samples obtained by the FeCl_3_/FeSO_4_ mixture with a ratio of Fe(iii) : Fe(ii) = 1 : 2 showed an irregular shape of the nanoparticles and high aggregation level. MNPs obtained by the FeCl_3_/FeSO_4_/CoCl_2_ mixture showed a regular shape with slight aggregation, and were in the nanosize range (10–17 nm). Thus, this mixture as a metal-precursor was used for MNP biosynthesis in the subsequent experiments. The XRD data showed that the magnetic specimens contained mainly spinel type phase. The data of EDX and XPS analysis indicated that the product of the “green” synthesis was magnetite with some impurities, owing to the obtained ratio of Fe : O being similar to the theoretical atomic ratio of magnetite (3 : 4). The Fe_3_O_4_-NP samples were superparamagnetic with high magnetization (until 68 emu g^−1^). The Co-containing MNPs demonstrated low ferromagnetic properties. The MNPs with pure magnetite phase, very good magnetization and uniform size distribution (*ca.* 12–14 nm) were prepared by the “hairy” root extract characterized by the highest amount of total flavonoids. According to the FTIR data, the synthesized Fe_3_O_4_-NPs had a core–shell like structure, in which the core was composed of Fe_3_O_4_, and the shell was formed by bioactive molecules. The presence of several organic compounds (such as flavonoids or carboxylic acids) plays a key role in the suppression of Fe_3_O_4_-NP aggregation without addition of a stabilizing agents. Synthesized Fe_3_O_4_-NP samples effectively removed Cu(ii) and Cd(ii) with the maximum adsorption capacity, reaching 29.9 mg g^−1^ and 33.5 mg g^−1^, respectively. It is probable that the presence of organic components in extracts plays an important role in the adsorption properties of biosynthesised MNPs. The obtained MNPs were successfully applied to the removal of heavy metal ions in the environmental water samples. Fe_3_O_4_-NPs also negatively affected plant growth in the case of using “hairy” roots as a test model, and the greatest inhibitory activity (99.56 wt%) was possessed by MNPs with high magnetic properties.

## Abstract 2

The “green” synthesis of magnetite and cobalt ferrite nanoparticles (Fe_3_O_4_-NPs and CoFe_2_O_4_-NPs) using extracts of *Artemisia annua* L “hairy” roots was proposed.

## Graphical abstract

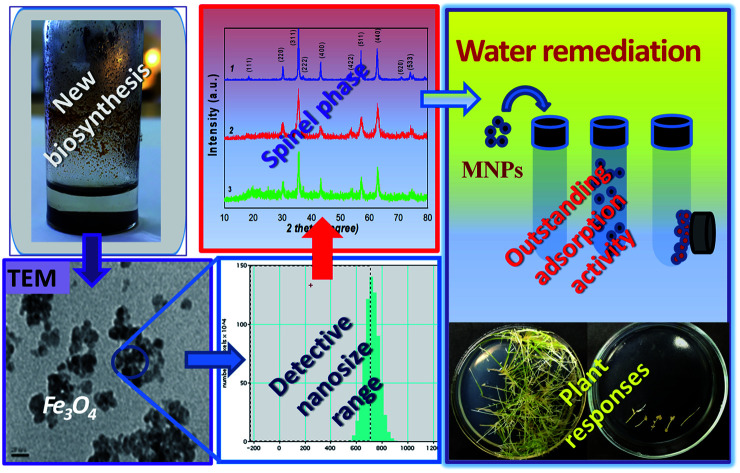

## 1. Introduction

The XXI century could be named as the century of “Nanotechnologies” because of the widespread use of special nanosize materials in different applications.^1,2^ The nanoparticles usually have other properties than bulk materials due to their smaller size, and are used as catalysts,^3,4^ in environmental analysis,^1,5,6^ in biology and in medicine.^2,7^ There are different groups of magnetic nanoparticles (MNPs) based on various chemical compositions, including pure metals (Fe, Co, and Ni),^3,5,8^ metal oxides (Fe_3_O_4_ (magnetite),^9,10^ α-Fe_2_O_3_ (hematite), γ-Fe_2_O_3_ (maghemite) and various iron oxyhydroxides (ferrihydrite, α-FeOOH (goethite)^4^)), ferrites (MFe_2_O_4_ (M = Cu, Ni, Co)), metallic nanocomposites^11^ and alloys (FePt, CoPt) with a wide range of nanoscale sizes. Iron oxides, such as magnetite (Fe_3_O_4_) and maghemite (γ-Fe_2_O_3_), are more prevalent because of the relatively good stability, magnetic moment and low toxicity. The chemical nature and the specific surface of ferric oxides make it efficient adsorbents for metal cations. Fe_3_O_4_ and γ-Fe_2_O_3_ magnetic nanomaterials have attracted attention in biomedical and pharmaceutical applications because of their biocompatibility, non-biodegradable and low toxicity for human cells.^12^

Various methods can be used for nanomaterials synthesis, like the sol–gel method, chemical reduction, co-precipitation, solvothermal/hydrothermal, microbial, and others.^13^ Application of nanotechnologies in biology initiated a swift increase of investigations in the use of plants for the synthesis of metal nanoparticles for their application in medicine and industry. Plant extracts possess reducing activity and due to this capacity, they can be used for the so-called “green” synthesis of various types of nanoparticles.^14^ There are several reviews about the methods of synthesis and practical application of “green” synthesized MNPs in comparison with nanoparticles obtained by “non-green” preparation.^13,15,16^ The reagents used in these “non-green” approaches are considered to be harmful for the environment. “Green” synthesized iron oxide nanoparticles were found to be nontoxic compared with ones prepared using chemical reduction agents.^17^ Thus, the MNPs obtained by ‘green’ synthesis have found their recent applications in wastewater treatment by the removal of toxic pollutants,^9,17,18^ in medicine as antimicrobial^19^ and anticancer agents,^5,8,20^ and also as a component in drug delivery systems, magnetic resonance imaging, bacterial detection, hyperthermia, and magnetofection.^11,21^ In the case of using “green” MNPs, the toxicity towards human organisms is minimal because plant extracts used for synthesizing Fe_3_O_4_-NPs are safe for the environment, and their use would thus be beneficial in biomedical applications.^11^ In addition, these MNPs can conjugate with drugs, enzymes or proteins, which can be directed to the targeted tissue, organ or tumor with the aid of an external magnetic field.

Different plants were used for obtaining extracts in order to prepare MNPs. For example, *Hordeum vulgare*,^22^*Yarrowia lipolytica*,^23^*Carum carvi*,^24^*Carica papaya*^4^ and others were studied for this purpose. For this aim, different parts of the plants (leaves, flowers, seeds, and fruits) were used.^4^ It should be noted that, in fact, the selection of plants was occasionally carried out by different authors. The green tea extract is most commonly used as a metal reducer for this purpose.^17,25^

Usually, the compositions of plant extracts are relatively complicated, and the concentration of natural components differs in various plants. At the same time, extraction conditions (solvent, temperature, *etc.*) also affect the extract composition. Therefore, there is little information about the key components in plant extracts that are crucial for the successful synthesis of MNPs with high yield. At the same time, numerous investigations of the last twenty years have revealed the great differences in the size, shape and activity of nanoparticles in the case of different plants used for the synthesis. That is why, in our opinion, the analyzing the “green” synthesis process for magnetic nanoparticles and evaluating the differences between the synthetic activities of the extracts are important steps to create a strategy for the most effective MNPs synthetic process.

In the reported studies, different iron salt combinations are used in the solutions for magnetic nanoparticles synthesis. FeCl_2_,^26^ FeCl_3_,^4,27^ Fe(NO_3_)_3_,^27^ FeSO_4_,^28^ and a combination of ferric and ferrous chloride^21^ salt solutions were studied to produce MNPs by “green” synthesis. Some factors affect the “green” synthesis process of MNPs. In particular, the heating time and temperature change the Fe_3_O_4_ yield. At the same time, this yield depends on the extract type used in the process,^26^ metal source,^27^ and ratio of the metal salt/extract. Chemical composition of plant extracts can play several functions. They take part in obtaining MNPs, but also act as stabilizers of these particles to suppress their aggregations. This occurs due to the presence of different components of the extract, such as ascorbic acid,^29^ amino acids,^30^ starch,^31^ glucose and gluconic acid,^27^ or the interaction of these products in the bioreaction mixture. So, the components of plant extracts are able to improve the properties of biosynthesized nanoparticles. Identifying the key components in this process is a very significant study. It can give additional information for finding the optimal conditions for the effective formation of MNPs with improved features.

We have recently reported a new approach to achieve size-controlled water-dispersible silver nanoparticles by “green” synthesis with *Artemisia annua* and *Artemisia tilesii* “hairy” root extracts.^32^ These water-dispersible biocompatible nanoparticles demonstrated antimicrobial properties. At the same time, they were stable as a suspension for a long time due to the presence of flavonoids and sugars in the extracts. These characteristics make them a potential new generation of NPs because saccharides also represent specific ligands for the synthesis and stabilization of MNPs. According to some reports,^33,34^ extract ingredients (such as reducing sugars, polyphenols, gallic acid and flavones) are the most effective for the synthesis of metal nanoparticles, including iron oxide. The content of these components varied from plant to plant. Our previous studies also demonstrated that polyphenols, gallic acid and flavones were the most active biomolecules in wormwood “hairy” root ethanol extracts.^32^ Due to the presence of the named components in “hairy” root extracts studied in our preliminary work, we would like to emphasize the possibility of using “hairy” root extracts for the synthesis of MNPs. It should be noted that a study in this direction seems to be very promising based on the increasing number of named components in “hairy” roots in comparison with the original mother plants. These features are associated with activating the synthesis of compounds with reducing activity in the cells of these roots after the incorporation of *rol* genes of *Agrobacterium rhizogenes*.^35^

In this paper, the possibility of a “green” and facile method for the synthesis of Fe_3_O_4_ and CoFe_2_O_4_-NPs using the ethanol extracts of *A. annua* “hairy” roots is reported. The synthetic potentials of the extracts of the mother (control) plants and the extracts from the transgenic (“hairy”) root lines are compared. The effect of the Fe(ii,iii) and Co(ii) salts mixture on the process of MNPs biosynthesis is evaluated. The prepared magnetic materials were tested for the plant inhibitory activities process. The adsorptive performance of the obtained MNPs was investigated for the removal of some heavy metals from environmental water samples.

## 2. Experimental section

### 2.1 Chemicals and raw materials

All reagents were of analytical grade and used as supplied. All solutions were prepared using distillate water. 96% ethanol was purchased from Sigma-Aldrich (HPLC grade). Iron(iii) chloride hexahydrate (FeCl_3_·6H_2_O, Merck), iron(ii) chloride tetrahydrate (FeCl_2_·4H_2_O, Merck), iron(ii) sulfate heptahydrate (FeSO_4_·7H_2_O, Alfa Aesar, ≥98%), copper nitrate (Cu(NO_3_)_2_·3H_2_O, Alfa Aesar, 99.999%) and ammonium hydroxide (NH_4_OH, Alfa Aesar, 25%) were used for sample preparation and treatment.

Stock solutions of Ni(ii), Cd(ii) and Zn(ii) ions were prepared by dissolving a weighed quantity of the respective nitrates or chlorides in distilled water. Stock solutions of Cu(ii) were prepared by dissolving calculated amounts of Cu(NO_3_)_2_·3H_2_O in 1 vol% HNO_3_. Working solutions were prepared by stepwise dilution with HNO_3_ (1 vol%). The ICP multi-element standard solution IV CertiPUR® Merck (CAS #111355, Supelco) for 23 elements (Ag, Al, B, Ba, Bi, Ca, Cd, Co, Cr, Cu, Fe, Ga, In, K, Li, Mg, Mn, Na, Ni, Pb, Sr, Tl, Zn, 1000 mg L^−1^ in nitric acid) was used for selectivity studies (Certificate of Analysis 1.11355.0100). HNO_3_ and NaOH were used to adjust the pH of the solution.

*A. annua* “hairy” root cultures from the collection of the Laboratory of Adaptational Biotechnology of the Institute of Cell Biology and Genetic Engineering NAS of Ukraine were grown under sterile conditions on Petri dishes using Murashige and Skoog solid nutrient medium (Duchefa, Netherlands) with twice reduced concentration (½MS). Mother plants, used as the control, were cultivated in the same conditions.

Transgenic and control lyophilized roots were used as the material to obtain extracts. Initially, 50 mg of dried leaves and roots of the control plants (samples no. 1 and no. 2) and “hairy” roots of *A. annua* (clones no. 3–6), were powdered by Retsch MM400, Germany, and added to 5 mL of 70 vol% EtOH for 3 days on the rotary shaker Clim-O-Shake system Kuhner IRC-1-U at 28 °C. Then, the extracts were centrifuged (Eppendorf centrifuge 5415 C, Germany) and supernatants were used for the synthesis of nanoparticles.

### 2.2 Synthesis and characterization of MNPs

#### The effect of Fe(ii,iii) and Co(ii) salts composition on the MNPs synthesis

For evaluation of the effect of the inorganic salts (FeCl_3_·6H_2_O – 5% (0.17 mmol L^−1^); FeSO_4_·7H_2_O – 2% (0.07 mmol L^−1^); FeCl_2_·4H_2_O – 5% (0.25 mmol L^−1^); CoCl_2_·2H_2_O – 2% (0.12 mmol L^−1^)) on the MNPs synthesis, the solutions were continually mixed in various ratios (Table 1) and the extract no. 4-1 was added at room temperature for 30 min. The pH was adjusted to 10 using ammonia solution (10%).

**Table 1:** The composition of the reaction mixtures used for MNPs biosynthesis

Mixture	Fe(iii) : Fe(ii)	Ratio in the reaction mixture, mol (mL)				Reaction mixture
		FeCl_3_	FeCl_2_	FeSO_4_	CoCl_2_	
**Divalent ions**						
No. 1	—	—	2.5(1)	1(0.72)	—	FeCl_2_ + FeSO_4_
No. 2	—	—	2.5(1)		0.12(0.1)	FeCl_2_ + FeSO_4_ + CoCl_2_

**Fe(** **iii** **,** **ii** **) and Co(** **ii** **) ions**						
No. 3	2 : 1	1.73(1)	—	1(0.72)	—	FeCl_3_ + FeSO_4_
No. 4	2 : 1	1.73(1)	—		0.12(0.1)	FeCl_3_ + FeSO_4_ + CoCl_2_
No. 5	1 : 2	0.87(0.5)	1.25(0.5)		—	FeCl_3_ + FeCl_2_ + FeSO_4_
No. 6	1 : 2	0.87(0.5)	1.25(0.5)		0.12(0.1)	FeCl_3_ + FeCl_2_ + FeSO_4_ + CoCl_2_

#### Synthesis of Fe_3_O_4_-NPs and CoFe_2_O_4_-NPs

70 vol% EtOH “hairy” root extracts (no. 1–6) were added to the salt mixture in the ratio of 0.5 mL of extract/2 mL of the salt solution. The pH was adjusted to 10 using 10% ammonia solution. Usually, synthesized samples were stored in ethanol prior to use.

For the characterization of MNPs, the mixture was magnetically separated to acquire solid black nanoparticles, which were then sequentially leached with deionized water once and ethanol thrice. The final solids were vacuum-dried for 24 h at 60 °C.

#### MNPs characterization

The powder X-ray diffraction (XRD) study of the obtained materials was carried out using a PANalytical X'Pert Pro diffractometer. Fourier transform infrared spectroscopy (FTIR) in KBr pellets was recorded using a Nicolet 470 Nexus instrument (Thermo Scientific, USA). Magnetic properties of nanoparticles were measured with an EV9 vibrating sample magnetometer. In order to determine the saturation magnetization (*M*_s_), magnetic hysteresis loop experiments were performed in a magnetic field (*H*) of 20 kOe. Transmission electron microscopy (TEM) was used to examine the size and morphology of the biosynthesized MNPs. The images were done by TEM 1230 JEOL (Tokyo, Japan) with an acceleration voltage of 80 kV. The JSM-6100 (JEOL, Japan) scanning electron microscope (SEM) equipped with an Oxford Instruments INCA energy-dispersive X-ray spectrometer (EDX) was used to study the chemical composition of solids. Textural parameters of the magnetic materials were determined from the N_2_ adsorption/desorption isotherms recorded at 77 K with an ASAP 2020 apparatus. The specific surface area (*S*_BET_) of the samples was determined by the BET method.^36^ X-ray photoelectron spectroscopy (XPS) spectra were measured with an Axis Ultra DLD electron spectrometer (Kratos Analytical, UK) using a monochromated Al Kα source that was operated at 150 W.

### 2.3 Flavonoid content assay

The total flavonoid content was studied by a slightly modified method using AlCl_3_ solution.^37^ 0.25 mL of each extract was mixed with 1 mL of double-distilled water and 0.075 mL of 5% NaNO_2_ solution. In 5 min, 0.075 mL of 10% AlCl_3_ solution was added to the reaction mixture. Then, 0.5 mL of 1 M NaOH solution and 0.6 mL of deionized water were added, and the absorbance of the samples was measured at 510 nm using a UV-Vis spectrophotometer (Fluorat-02 Panorama, Russia). The total flavonoid content was calculated by the calibration plot: *A*_510_ = 0.09002*C*_rutin_ − 0.0309 (*R*^2^ = 0.9996) and expressed as milligrams per gram of dry root weight in rutin equivalent (mg RE per g DW).

### 2.4 Reducing power assay

The reducing power, as the ability of extracts to reduce ferric Fe^3+^ ions, was measured according to the method described by Chan *et al.*^38^ The extracts of the different amounts (0.016–0.125 mL) were added to 0.312 mL of 0.2 M phosphate buffer (pH 6.6) and 0.312 mL of 1% potassium ferricyanide, mixed thoroughly and incubated at 50 °C for 30 min. After this procedure, 0.312 mL of 10% trichloroacetic acid solution was added. Then, 1.25 mL of the solutions was mixed with 1.25 mL of deionised water and 0.25 mL of 0.1% ferric chloride. The absorbance was spectrophotometrically studied at 700 nm (Fluorat-02 Panorama, Russia). The reducing power expressed as equivalent concentrations (EC_0.5_) was determined as the amount of dried root material needed to obtain an absorbance value of *A*_700_ = 0.5. This parameter was calculated by the equations: *A*_700_ = *f*(*C*_extract_). Rutin solution was used as a positive control.

### 2.5 Statistical analysis

All analyses were carried out in triplicate. Data were expressed as the mean and confidence interval (CI) at the 95% level. The data were analyzed for statistical significance using ANOVA, followed by Tukey HSD test. *P* values less than 0.05 were considered significant. The linear regression method was applied, and the coefficient of determination (*R*^2^) was calculated for establishing the relationship between the values.

### 2.6 Estimation of Fe_3_O_4_-NPs toxicity to plants

*Althaea officinalis* “hairy” root culture was used as an *in vitro* model to determine the toxicity of MNPs. The roots were grown *in vitro* on Petri dishes using Murashige and Skoog solid nutrient medium (Duchefa, Netherlands) with twice reduced concentration. Nanoparticles (40 μL) were added to 10 mL of medium 1/2MS. Root tips (10 mm long) were planted on the surface of the medium and grown during 3 weeks at a temperature of +25 °C. The inhibition (*I*, %) effect was calculated using the below formula:
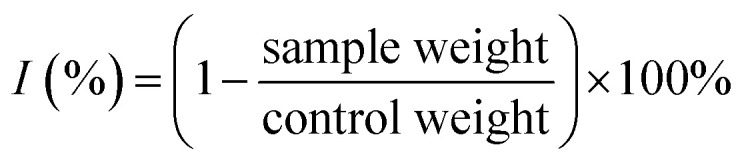


### 2.7 Batch adsorption experiments

Batch experiments were used to determine the efficiency of MNPs to remove metal ions from aqueous solution. Briefly, MNPs (0.05 g) were weighed into a 13 mL centrifuge tube, and a 10 mg L^−1^ Cu(ii) solution (10 mL) was added. The reaction solution was continuously stirred at a constant temperature (20 °C) without any pH adjustment. The solution was magnetically separated prior to the determination of the residual metal ion concentration.

The residual concentration of metal ions in solutions after the adsorption test was determined by atomic absorption spectrometry (AAS) on an Analyst 3030 (PerkinElmer) spectrometer with acetylene-air flame atomization: Cu (324.75 nm), Zn (213.86 nm), Cd (228.80 nm), Ca (422.67 nm), Pb (217.00 nm) and Fe (248.33 nm).

#### Adsorption isotherms

The isotherm adsorption of the metal ions was studied at room temperature under static conditions at constant media pH, particularly at pH 4.5 (Cu), 5.2 (Zn) and 4.8 (Cd). Typically, 30 mg of MNPs was added to 20 mL of metal ions solution within the 20–230 mg L^−1^ concentration, and mechanically stirred for 30 min. After that, the adsorbent was magnetically separated, and the remaining concentration of the metals in solution was determined by AAS.

The adsorption capacity (*q*_e_, mg g^−1^) and recovery (*R*, %) of MNPs toward metal ions was calculated using the following equations:
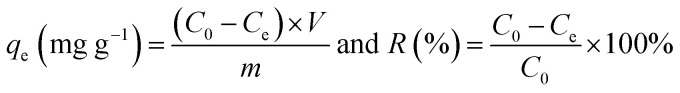
and where: *q*_e_ is the amount of metals adsorbed per mass unit of adsorbent at equilibrium (mg g^−1^); *C*_0_ and *C*_e_ are the initial and equilibrium concentrations of the metal ions (mg dm^−3^), respectively; *V* is the volume of the solution (dm^3^), and *m* is the weight of the solid (g).

#### Sample preparation for water purification

River water samples were collected from the Dnipro River (Kyiv, Ukraine) in June 2021, and stored at 4 °C after conservation with HNO_3_. Before the experimental procedure, river water samples were filtered through a membrane filter (Millipore, 0.45 μm). Then, 30 mg of MNPs was added to 200 mL of the river water after pH adjusting and agitated for 30 min. All of the samples after the extraction procedure were subsequently measured by AAS.

## 3. Results and its discussions

### 3.1 “Hairy” roots extract preparation and characterization

**Fig. 1:**
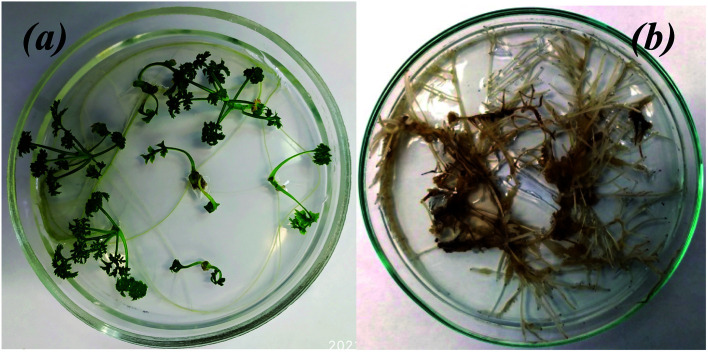
*Artemisia annua* control plants (a) and “hairy” roots culture (b) grown *in vitro*.

Control plants and “hairy” roots of *Artemisia annua* were cultivated *in vitro* under the same conditions (medium composition and temperature) (Fig. 1).

**Fig. 2:**
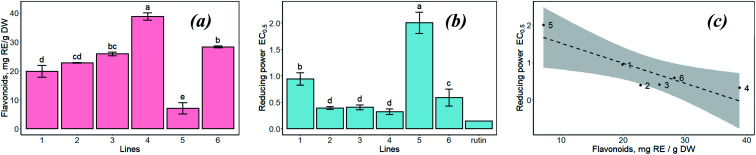
Total flavonoid content (a), reducing power (evaluated as EC_0.5_) (b) and their correlation (c) in EtOH extracts of *A. annua* control leaves (no. 1), roots (no. 2), and “hairy” root lines (no. 3–6).

To assess the key parameters of extracts for MNP biosynthesis, the level of flavonoids accumulation and reducing power of extracts from the control plants and “hairy” roots were studied. In some cases, the total flavonoid content in the extracts obtained from the “hairy” roots exceeded the same parameter in the control roots and leaves (Fig. 2a). In particular, the total flavonoid content in transgenic root line no. 4 was greater than that in the control roots (38.8 ± 3.09 and 22.7 ± 0.27 mg g^−1^ DW, respectively).

The results of the reducing power assay indicate that all extracts tested had low activity compared to rutin (1 mg mL^−1^) (Fig. 2b). In the case of “hairy” roots extracts, the lowest value of reducing power it showed by no. 5 sample. In general, the extracts of “hairy” root samples (except for no. 5) showed higher reducing power than the extracts of the control samples. The reducing power calculated as a lower EC_0.5_ value indicated in the “hairy” root clones was correlated with the total flavonoids content (Fig. 2c) with the following regression equation: *y* = 2.050 − 0.0534*x*, *R*^2^ = 0.758, CI = 0.95. Deviations from the regression plot can be explained by the presence of other reducing agents in the extract.

Consistent with the obtained results, the “hairy” root extracts can be used as effective agents for the synthesis of various nanoparticles of different morphology because they contained a high amount of flavonoids and other organic compounds.^32^

### 3.2 Optimization of MNPs synthesis

The possibility of MNPs synthesis using EtOH extract no. 4 of the “hairy” root lines *Artemisia annua* (with high total flavonoid content) was evaluated. The biosynthesis procedure of MNPs was optimized by metal ions precursors, particle size and morphology using transmission electron microscopy (TEM).

**Fig. 3:**
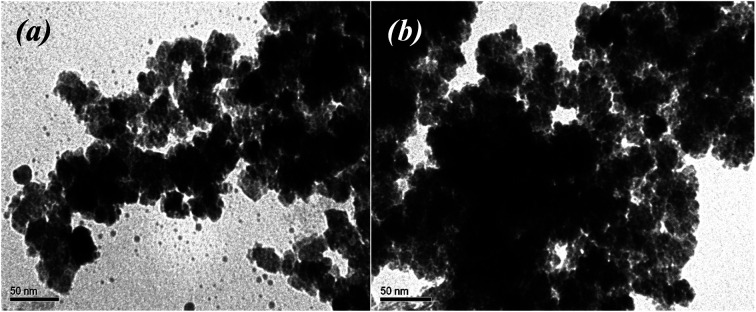
TEM images of nanoparticles obtained by using Fe(ii) and Co(ii) salts (a – mixture no. 1 (FeCl_2_ + FeSO_4_), b – mixture no. 2 (FeCl_2_ + FeSO_4_ + CoCl_2_)).

In the first stage, the effect of the oxidation state and metal salt precursor(s) in these parameters has been studied. For producing MNPs, two different approaches were used:

(a) Biosynthesis of MNPs using only divalent salts of metal precursors with various anions in the reaction mixture (Fig. 3);

According to the TEM data, MNPs were successfully synthesized using divalent salts of metal ions containing Fe(ii) and Co(ii) cations (Fig. 3). In this process, it is probable that the formation of Fe_3_O_4_-NPs (or CoFe_2_O_4_-NPs) can be described *via* the following steps:^13,39^Fe^2+^ + 2OH^−^ → Fe(OH)_2_4Fe(OH)_2_ + O_2_ → 4FeOOH + 2H_2_O2FeOOH + Fe(OH)_2_ + 4OH^−^ → Fe_3_O_4_↓_(solid)_ + 4H_2_Oor in the case of the presence of Co(ii) in the reaction mixture:Co^2+^ + 2OH^−^ → Co(OH)_2_2FeOOH + Co(OH)_2_ + 4OH^−^ → CoFe_2_O_4_↓_(solid)_ + 4H_2_O

To minimize the overall energy of the MNPs on the suspension, the small nanoparticles tended to aggregate and form larger MNPs. It was found that electron-dense spherical and non-spherical nanoparticles with a diameter of up to 20 nm were formed. However, when the FeCl_2_ + FeSO_4_ salts combination was used, precipitation occurred and the solution turned colorless with large dispersed particles. The particle size is an important property, such as when the nanoparticles are used for pharmaceutical and biomedical purposes.^12^ In addition, MNPs that are oxidizable by environmental oxygen easily provoke a significant reduction of their magnetism and dispersibility.^40^ When the reaction mixture contained CoCl_2_, the MNPs were uniform and smaller than the particles obtained using a reaction mixture containing only mono iron(ii) salt (10.5–16.0 nm) (Fig. 3b). The aggregation effect of MNPs obtained from the FeCl_2_ + FeSO_4_ + CoCl_2_ mixture was isotropic in each direction. Nevertheless, agglomerations of MNPs can locally increase the amount of iron ions, causing toxicity to human health. For future therapeutic or biological application of biosynthesized MNPS, it is necessary to improve its dispensability in water with low aggregation.

**Fig. 4:**
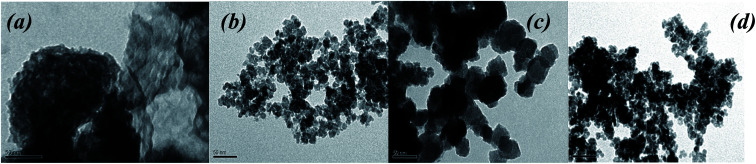
TEM images of MNPs by using Fe(ii,iii) and Co(ii) salts: a – no. 3 mixture (FeCl_3_ + FeSO_4_), b – no. 4 mixture (FeCl_3_ + FeSO_4_ + CoCl_2_); c – no. 5 mixture (FeCl_3_ + FeCl_2_ + FeSO_4_), d – no. 6 mixture (FeCl_3_ + FeCl_2_ + FeSO_4_ + CoCl_2_).

(b) Biosynthesis of MNPs using inorganic salts with various oxidation states and different anions. The various ratios of Fe(iii) : Fe(ii) were also studied to understand the mechanism of MNPs formation (Table 1). Effects of these parameters on the morphology and size were controlled by TEM (Fig. 4).

The addition of “hairy” root extract in all cases successfully resulted in nanoparticle formation (Fig. 4). TEM images indicated that the nanoparticles were quite round and irregular in shape, and polydispersed with a high size distribution. These features depended on the reaction mixture components. Therefore, the results showed that the morphology and particle size in the process of “green” MNPs formation depended on the ratio of Fe(iii) : Fe(ii) in the initial reaction mixture. Samples obtained in the no. 3 mixture (FeCl_3_ + FeSO_4_) with a ratio of Fe(iii) : Fe(ii) = 2 : 1 showed an irregular shape of nanoparticles and high aggregation level (Fig. 4a). MNPs obtained from the FeCl_3_ + FeSO_4_ + CoCl_2_ reaction mixture (Fig. 4b) were very regular, slightly aggregated and had a particle size ranging from 10 to 17 nm, with an average of 13.5 nm. MNPs obtained from the FeCl_3_ + FeCl_2_ + FeSO_4_ mixture (Fig. 4c) were much greater, with particle sizes ranging from 20 to 50 nm (average of 31.92 nm) with a cubic structure, and lower size distribution nanoparticles were obtained by using the FeCl_3_ + FeCl_2_ + FeSO_4_ + CoCl_2_ mixture (Fig. 4d).

Based on the results, the reaction no. 4 mixture containing FeCl_3_ + FeSO_4_ + CoCl_2_ salts with a ratio of Fe(iii) : Fe(ii) = 2 : 1 was selected as better for the next experiments to obtain MNPs using different *Artemisia annua* extracts (control non-transgenic leaves, and roots and “hairy” roots).

### 3.3 Synthesis and characterization of MNPs in optimal conditions

In the second stage, EtOH extracts of the control *A. annua* plants and the four “hairy” root lines were used for MNPs synthesis with the FeCl_3_ + FeSO_4_ + CoCl_2_ mixture.

**Fig. 5:**
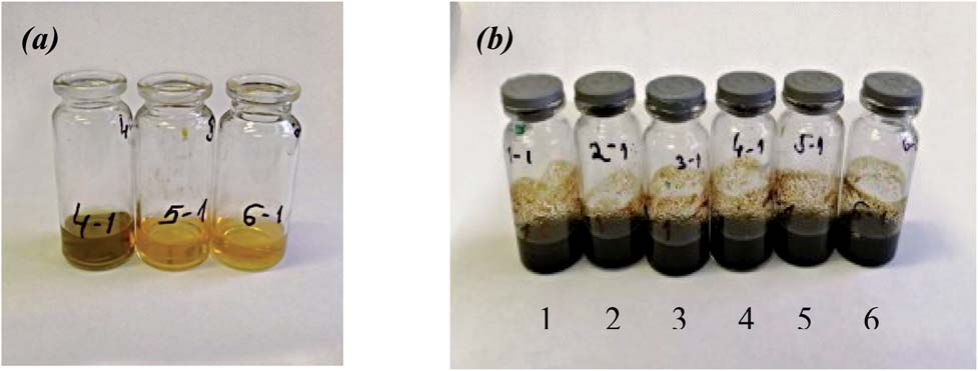
Color change after the addition of the extracts of *Artemisia annua* control leaves (1) and roots (2), and “hairy” roots (3–6) to the FeCl_3_ + FeSO_4_ + CoCl_2_ mixture with pH 3 (a) and 9 (b).

The addition of ethanol extracts of *A. annua* to the mixture of Fe(iii) and Fe(ii) salts resulted in the solution color changing from yellow to intense brown with dark precipitates, depending on the pH mixture (Fig. 5). The color change from yellow to dark and magnet-filling effect at separation indicated the formation of MNPs. These phenotypic changes of the mixture solutions correlated well with the total flavonoid content data (Fig. 2a). It should be emphasized that when we used the extract no. 4 characterized by the highest flavonoid content and great reducing power, the color change of the solution occurred almost immediately from yellow to brown after the addition of ferric salts without increasing the pH to 10. Browning of the solutions in the case of adding other extracts was observed only at pH 10 (Fig. 5a).

This effect was not observed in other studies conducted to produce Fe_3_O_4_-NPs by chemical co-precipitation (Massart) method, according to the following reaction:^13^Fe^2+^ + 2Fe^3+^ + 8NH_4_OH → Fe_3_O_4(solid)_↓ + 8NH_4_^−^ + 4H_2_O

### 3.4 MNPs characteristics

**Fig. 6:**
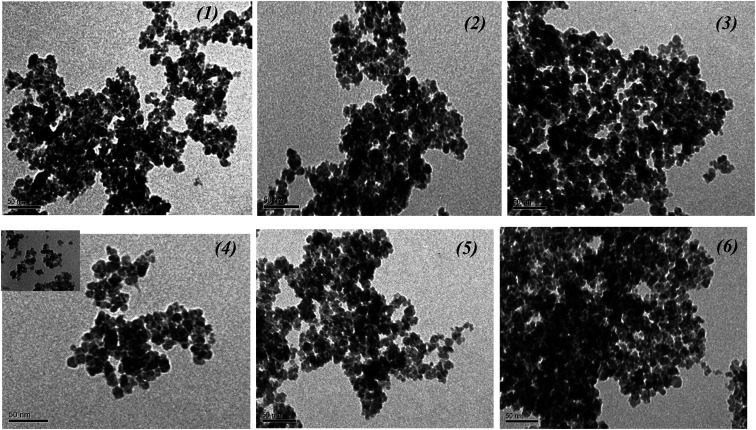
TEM images of Fe_3_O_4_-NPs obtained with extracts of the control leaves (no. 1), roots (no. 2) and “hairy” roots of *Artemisia annua*” (no. 3–6) after the addition to the FeCl_3_ + FeSO_4_ + CoCl_2_ mixture.

The morphology of the biosynthesized nanoparticles was visualized using TEM. We used the salt mixture (FeCl_3_ + FeSO_4_ + CoCl_2_, Table 1), added the extracts of the control leaves and roots, and also “hairy” root extracts for MNPs synthesis. The TEM images demonstrated the differences in the shape and size of the MNPs (Fig. 6).

**Fig. 7:**
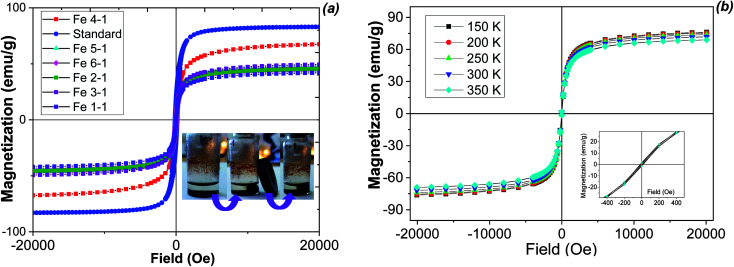
Wide magnetic hysteresis loops and magnetic separation (inset) of biosynthesized MNPs at room temperature (a) and at different temperatures for the Fe 4-1 sample (b).

The magnetic behaviour of the MNPs was studied for all samples at room temperature. Then, the temperature dependence of the magnetization for the selected samples was measured (Fig. 7).

Magnetization curves measured at room temperature (Fig. 7a) for all samples saturates from 41.8 emu g^−1^ to 68.0 emu g^−1^. The difference of the saturation magnetization (*M*_s_) between MNPs obtained from various extracts is most likely attributed to the effect of the extract components. The magnetization saturation (*M*_s_) of the biosynthesized MNPs was smaller than that of the bulk magnetite (92 emu g^−1^, Fig. 7a), but it was similar to magnetite nanoparticles obtained by chemical co-precipitation method (48 emu g^−1^).^41^ The magnetic properties of the obtained MNPs were essentially different, indicating significant influence of the nature of the plant extract on the physical characteristics of the resulting solids. The high value of *M*_s_ is commonly attributed to the stabilization effect of the extract components. The highest magnetization saturation was observed for the Fe 4-1 sample (68.0 emu g^−1^). This parameter was about 23% higher than the same characteristic of other MNPs, and may be associated with the high reducing power and high amount of flavonoids of the extracts used for the synthesis of the Fe 4-1 sample (Fig. 2). In this sense, the flavonoids coating weakens the interaction between the nanoparticles, which in turns reduces the aggregation of the MNPs, thus increasing the magnetization saturation. Also, all of the samples had zero coercivity at 300 K, indicating essential superparamagnetic properties of the obtained Fe_3_O_4_-NPs.^42^ This is probably due to the crystallite sizes of MNPs, which were in the nanometer size range, according to the TEM data. Only the Fe 5-1 sample exhibited low hysteresis, which indicates the presence of ferromagnetism. Therefore, the particle sizes with high magnetization values and loss of magnetization after magnetic field removal are an important property for pharmaceutical and biomedical purposes.^43^ In addition, MNPs must combine high magnetic susceptibility for optimal magnetic enrichment and loss of magnetization after magnetic field removal.

For understanding the magnetic behaviour of biosynthesized MNPs, the temperature dependence of magnetizations at 150–350 K for the selective magnetic sample is shown in Fig. 7b. The low temperature (150 K) magnetization data also reveal that sample saturates at 68.0 emu g^−1^ with zero coercivity. The coercivity and remanence in the hysteresis loop for the MNPs demonstrated low decrease with the increase of the temperature, thus showing a superparamagnetic behaviour at all temperature range.

**Fig. 8:**
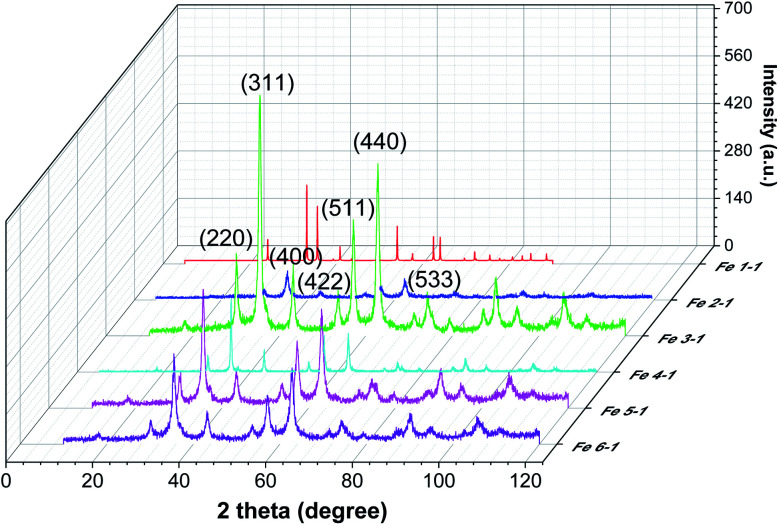
Powder XRD patterns of MNPs samples obtained using control leaves (Fe 1-1 sample), roots extracts (Fe 2-1 sample), and “hairy” roots extracts (Fe 3-1, Fe 4-1, Fe 5-1 and Fe 6-1 samples).

Phases identification of the prepared magnetic materials has been conducted using powder X-ray diffraction (Fig. 8).

It is obvious from the XRD patterns (Fig. 8) that there were some characteristic peaks at 2*θ* = 30.1°, 35.5°, 43.1°, 53.4°, 57°, 63.1°, and 74.9°, which related to the corresponding indices (220), (311), (400), (422), (511), (440) and (533), respectively. This indicates that the resulting MNPs were Fe_3_O_4_ (JCPDS no. 19-0629) with a face-centred cubic spinel crystalline structure that indexed with cell constant *a* = *b* = *c* = 8.375 Å. Moreover, the small peaks at 2*θ* = 21.22° (104) indicates that the goethite (α-FeOOH) phase was presented in the synthesized Fe 1-1 sample, which could not be discriminated because of the significant line broadening due to the small size of the crystalline domains. As expected, the line broadening increased upon decreasing the nanocrystal size. The line positions of the synthesized Fe 5-1 samples were indexed to the cubic phase of cobalt ferrite. The estimated lattice parameter *a* = 8.388 Å agrees well with the JCPDS card no. 22-1086. Thus, the modification of the reaction mixture by using various extracts changed the crystalline phase of MNPs. Also, the difference in the phase composition of the series of magnetic samples indicated that the oxidation of magnetite into maghemite was completed, and maghemite was reduced under the ‘green’ synthesis conditions to hematite. The average crystallite sizes calculated using Scherrer equation^44^ for the (311) reflection are 12.2 nm, 17.9 nm, 12.3 nm, 15,4 nm, 17,8 and 19.6 nm for the Fe 1-1, Fe 2-1, Fe 3-1, Fe 4-1, Fe 5-1 and Fe 6-1 samples, respectively. The crystallite size of the synthesized MNPs studied by XRD analysis is in agreement with the result obtained from TEM (Fig. 6).

The XRD patterns of the Fe_3_O_4_-NPs samples with various washing and drying conditions were studied (Fig. S1†), revealing that the structure was dramatically influenced by pre-treatment conditions. Appearance of the “halo” at 2*θ* = 20–30° of the as-prepared MNPs confirmed the formation of an amorphous phase. This was probably due to the reaction of MNPs *via* electrostatic interaction with organic components in the extract, which acted as an amorphous layer.

**Fig. 9:**
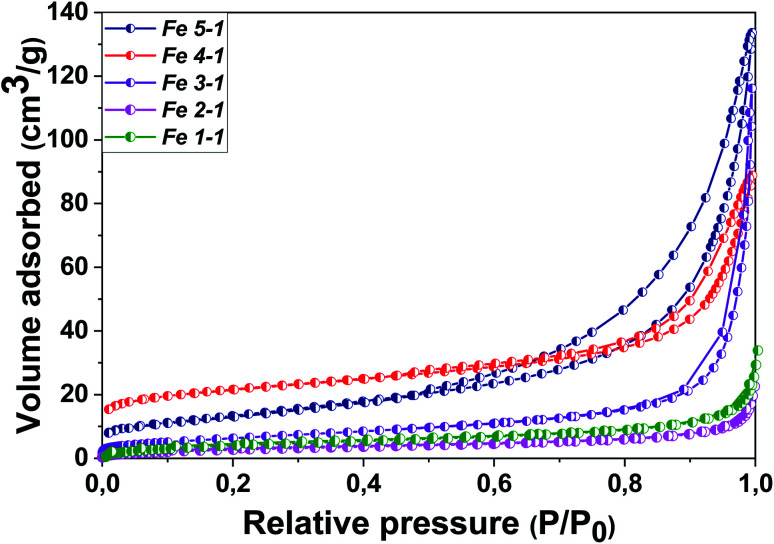
N_2_ adsorption/desorption isotherms for the as-synthesized Fe_3_O_4_-NPs.

The N_2_ isotherm adsorption/desorption of the Fe 1-1 and Fe 2-1 samples exhibit type I without hysteresis loops, according to the IUPAC classification,^45^ which is typical for microporous materials (Fig. 9). It was found that the Fe_3_O_4_-NPs samples obtained with “hairy” root extract have a micro–mesoporous space structure with an average pore size of 1.7–1.9 nm in diameter, although the mesopore contribution for most materials is insignificant. Thus, the “hairy” root extracts used in the MNPs synthesis resulted in an increase of porosity and specific surface areas (*S*_BET_).

### 3.5 Chemical composition of MNPs

**Fig. 10:**
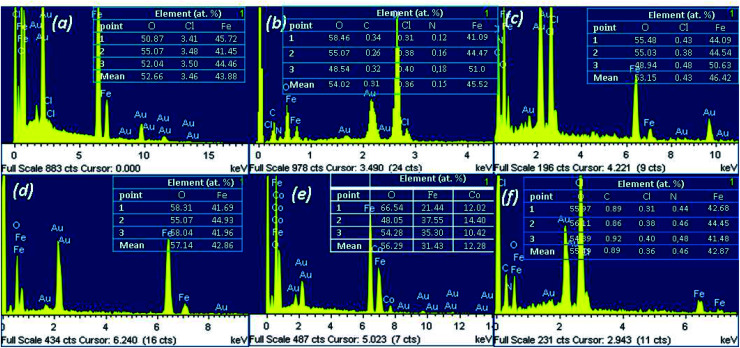
EDX spectra combined with microanalysis of the as-prepared MNPs: a – Fe 1-1; b – Fe 2-1; c – Fe 3-1; d – Fe 4-1, e – Fe 5-1 and f – Fe 6-1 samples.

Furthermore, the elemental composition of MNPs was characterized by SEM-EDX method. Fig. 10 shows the representative EDX spectra (1 spectrum from 3 point of analysis) of the MNPs. In these spectra, the peaks of Au were observed due to the gold-covering process of analysis.

The MNPs were primarily composed of iron (*K*_α_ at 6.4 keV) and oxygen (*K*_α_ at 0.6 keV). The spectra also reveal the presence of residual chloride ion impurities (Fig. 10a). Moreover, the EDX spectra sometimes depicted the signal of carbon and nitrogen, which were attached to Fe_3_O_4_-NPs due to the presence of bioactive compounds in the extracts. Although the EDX pattern is similar to the results presented in other reports,^41^ there were slight differences in the element intensities, which are likely related to variations in the valence of the iron species, synthesized under different conditions. EDX is a semi-quantitative technique and we could use it to determine the atomic ratios of Fe : O, which were about 2.9 : 3.48, 3,0 : 3.56, 3.1 : 3.55, 3 : 3.90, 2.2 : 3.78 and 2.92 : 3.78 for the Fe 1-1, Fe 2-1, Fe 3-1, Fe 4-1 and Fe 6-1 samples, respectively. The data indicated that the MNPs product was magnetite with some impurities, because the obtained ratio of Fe : O was similar to the theoretical atomic ratio of magnetite (3 : 4). These small difference atomic ratios of Fe : O in the obtained MNPs compared to bare magnetite was probably due to the formation of an impurity in the Fe_2_O_3_ phase (when freshly prepared Fe_3_O_4_-NPs were oxidized *via* reaction with oxygen in the air) or the present organic component in the MNPs surface. These hypotheses were discussed in the XRD characterizations of the MNPs and the morphology of the nanoparticles from the TEM images.

EDX spectra of the Fe 5-1 sample have shown peaks of Co, in addition to stronger signals of Fe (Fig. 10b). It was found that the Fe : Co molar ratio was ∼2 : 1. The result demonstrated that this sample is cobalt ferrite (CoFe_2_O_4_-NPs), confirmed by powder XRD data (Fig. 8). Thus, these results were probably due to the effect of a low concentration of flavonoids in the extract used for the synthesis of the Fe 5-1 nanoparticles.

The XPS provided information about the chemical composition and oxidation state of elements on the as-synthesized MNPs surface (Fig. S2†). The XPS survey spectra of Fe_3_O_4_-NPs show strong bands due to iron at 724 eV (Fe 2p_1/2_), 709 eV (Fe 2p_3/2_) and 60 eV (Fe 3p) (Fig. S2a†). In all cases, O 1s (532 eV) and N 1s (405 eV) features are seen in addition to small bands that are due to C 1s (≈285 eV, ≈2–7 at%), suggesting the presence of N,O-containing compounds on the MNPs surface. Indeed, the high resolution Fe 2p core level spectrum is shown next to the narrow and strong band at 709.5 eV (Fe 2p_3/2_) and the weaker band at 722.6 eV (Fe 2p_1/2_) with additional satellite peaks (Fig. S2b†). The peak position and the presence of the satellite peaks are typical for Fe_2_O_3_. The Fe/O ratios were 0.71, 0.72, 0.73, 0.73, 0,75 and 0.73 for the Fe 1-1, Fe 2-1, Fe 3-1, Fe 4-1 and Fe 6-1 samples, being somewhere in-between that of Fe_3_O_4_ (0.75) and Fe_2_O_3_ (0.66), indicating that some magnetic Fe_3_O_4_ was oxidized into Fe_2_O_3_.

FTIR spectroscopy was carried out to determine the extract-derived bioactive compounds that may coat the MNPs. The Fe_3_O_4_-NPs under different washing and drying conditions were analyzed by FTIR spectra.

**Fig. 11:**
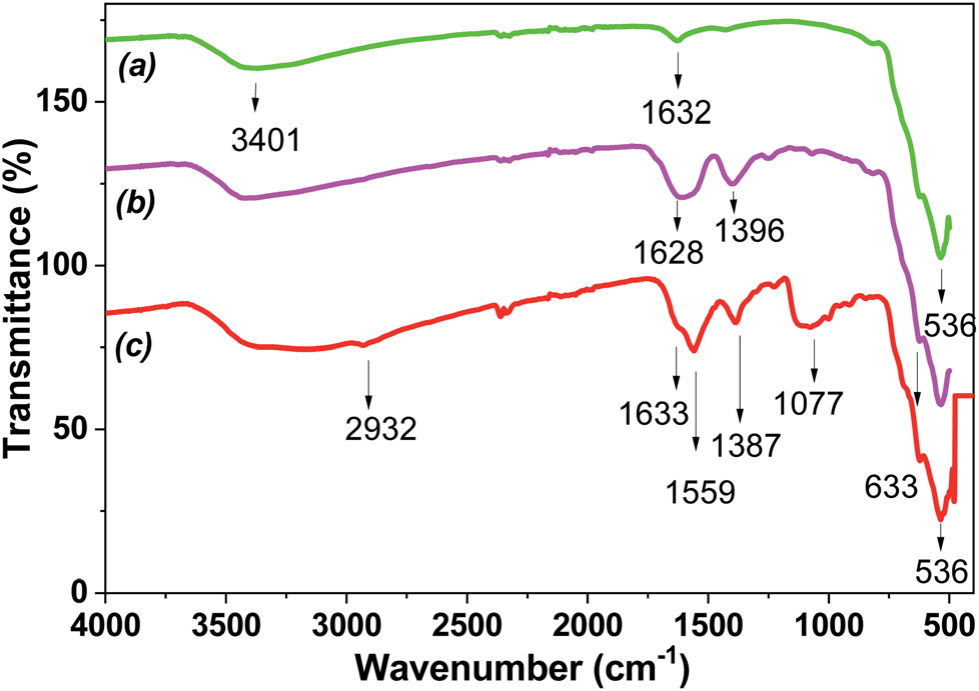
FTIR spectra of Fe_3_O_4_-NPs (Fe 4-1 sample): a – washing by water and dried in vacuum; b – washing by water and dried in air at 25 °C; c – dried in air at 25 °C.

Fig. 11 shows the FTIR spectra of the biosynthesized MNPs. The occurrence of the Fe–O bond about 536 cm^−1^ confirmed the formation of iron oxide in the biosynthesized MNPs.^41^ The absorption peak was about 820 cm^−1^ and 1315 cm^−1^, and assigned to the C–O–C and aromatic C–OH stretching bonds, respectively. The stretching vibrations at 1630 cm^−1^ and 3400 cm^−1^ corresponding to polyphenols were also observed in MNPs. The occurrence of stretching vibrations of polyphenols in MNPs confirmed the capping activity of the magnetite surface by polyphenols. The FTIR spectra of dried Fe_3_O_4_-NPs in extract exhibited strong peaks in the range of 1630 cm^−1^ and 3400 cm^−1^, corresponding to the alkene (C=C) stretching vibrations and phenolic hydroxyl groups (–OH), respectively. The data indicated the presence of hydrogen bonding between the extract-derived polyphenols.^32^ The shifts in peak position in the range of 400–4000 cm^−1^ ensured that these functional groups of the bioactive organic compounds can be bound to the iron oxide surface. Thus, the presence of wide spectra organic compounds in the extract played a key role in the formation of the Fe_3_O_4_-NPs core–shell structure, in which the core was composed of Fe_3_O_4_. At the same time, the core had the layer coating of bioactive compounds from “hairy” root extracts.

Thus, the XRD, XPS and FTIR results supported the surface of MNPs being covered with biomolecules.

### 3.6 Mechanism of MNPs formation by “hairy” root extracts

**Scheme 1:**
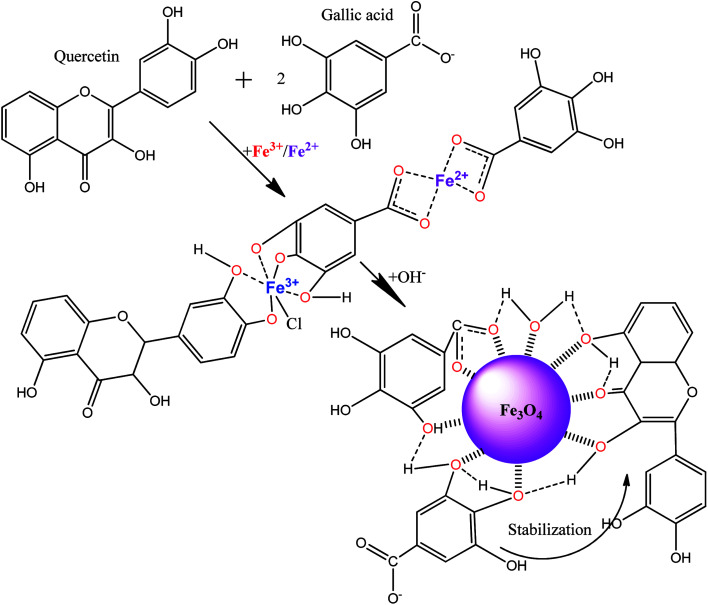
Schematic of the probable mechanisms of formation for MNPs by “hairy” root extract.

Considering the obtained results from the analysis of extracts and magnetic samples, a mechanism formation involving the core–shell MNPs structure from “hairy” roots extract is proposed (Scheme 1).

We suggest that polyphenols (for example, quercetin) and phenolcarboxylic acid (for example, gallic acid), which are abundant in the ethanol extract, can be responsible for the production of MNPs. Bioactive molecules and Fe(ii,iii) ions are the most important reagents involved in the reaction of the formation of Fe^3+^-polyphenol/phenolcarboxylic acid intermediate (Scheme 1). These complex compounds could stabilize MNPs during co-precipitation by OH^−^ without reduction of Fe(ii,iii) ions, and MNPs would be coated by bioactive-derivate compounds as a result. It was discovered that the MNPs suspensions studied are stable without aggregation for at least 6 months. The complexation reaction between the major components of the extract and Fe(ii,iii) salts is the first stage the ‘green’ synthesis. In the second stage, during a potentially much slower reaction, the other bioactive organic coordination compounds in the solution would continuously diffuse into the solid–liquid interface formed at the MNPs core surface. Thus, the surface of the MNPs coated by the extract-derived compounds additionally would be covered by adsorption or precipitation with other organic compounds from the extract.

### 3.7 Bioactivity of MNPs

To determine the effect of MNPs on the plants, *Althaea officinalis* “hairy” roots were used as an *in vitro* model to evaluate the probable toxicity of the MNPs on biosystem. MNPs were added to the medium for roots cultivation (Fig. S3† and Table 2).

**Table 2:** Inhibition effect of MNPs to the growth medium *in vitro* study on root weight (per one root tip)

	Sample	Weight, g	*I*, %
1	Without MNPs	0.682 ± 0.043	—
2	Fe 1-1	0.096 ± 0.019	85.92
3	Fe 2-1	0.182 ± 0.006	73.31
4	Fe 3-1	0.287 ± 0.012	57.92
5	Fe 4-1	0.003 ± 0.002	99.56
6	Fe 5-1	0.450 ± 0.017	34.02
7	Fe 6-1	0.582 ± 0.008	14.66

All MNPs prepared by biosynthesis inhibited the growth of “hairy” roots (Fig. S2b–g†), while the Fe 4-1 sample demonstrated the most toxic effect for roots growth (Table 2). It is of great interest that the most toxic effect on roots growth was found in the case of adding to the medium size nanoparticles obtained using “hairy” root extract Fe 4-1 with the highest reducing power and high flavonoids concentration.

### 3.8 Adsorption future of obtained MNPs

In order to examine the adsorption performance of the obtained MNPs, sorption of several heavy metal ions from water solutions was investigated in batch experiments. The effect of pH in the range of 3–7 on the adsorption of metal ions by bare magnetite was studied.^41^

#### Effect of pretreatment conditions on Cu(ii) removal by MNPs

**Fig. 12:**
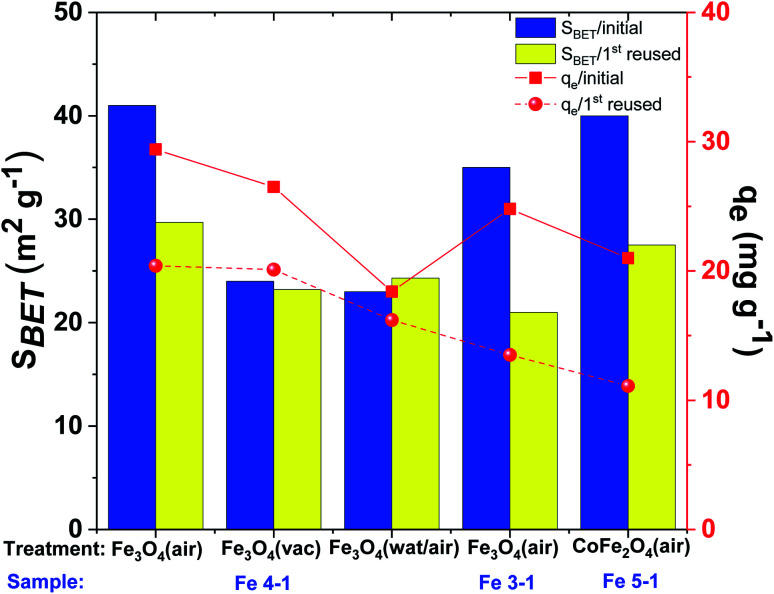
Comparative adsorption capacity (*q*_e_) and surface area (*S*_BET_) of Cu(ii) ions onto various pre-treatment of MNPs (C(Cu(ii)): 50 mg L^−1^, adsorbent dose: 50 mg, volume 15 mL, pH 4.5).

The effects of the pretreatment conditions of solids on the adsorption capacity and surface area are crucial to the features of MNPs as adsorbents for water treatment (Fig. 12). Cu(ii) ions are useful as a model analyte for easy comparison with other reported MNPs. Removal of Cu(ii) on Fe_3_O_4_ was conducted at pH 4.5 because of the minimum influence co-precipitation of Cu(OH)_2_ on the surface of MNPs at these conditions, while their uptake in this case is most effective.

The removal efficiency of Cu(ii) also dramatically depended on the conditions of Fe_3_O_4_-NPs pretreatment (Fig. 12). The maximum copper(ii) removal efficiency was shown when MNPs were simply dried without additional washing. Thus, synthesized Fe_3_O_4_-NPs had an organic bioactive shell-like layer, which have effect of ligands for target metal ions. The removal efficiency (50% removal overnight) was decreased when MNPs were washed with distillate water (or EtOH) and dried at room temperature in air. This effect occur probably due to the high degree of nanoparticles aggregation, which lead to decreased surface areas and reactivity to Cu(ii) ions. The removal efficiency of Fe_3_O_4_-NPs dried at 60 °C under vacuum was decreased. It was hypothesized that organic matter on the MNPs surface was more volatilized after thermal and vacuum treatments, confirmed by FTIR spectra (Fig. 11). Thus, the amount of active centers on the surface of the Fe_3_O_4_-NPs samples was significantly decreased.

#### Adsorption isotherms

**Fig. 13:**
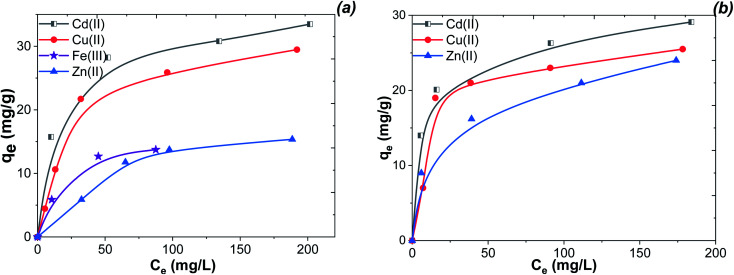
Isotherm adsorption of Fe 4-1 (a) and Fe 5-1 (b) samples towards Zn(ii), Cu(ii), Fe(iii) and Cd(ii) ions at pH 1.8 (Fe), 4.8 (Cd), 5.2 (Zn), and 4.5 (Cu) (conditions: initial metal concentration 20–230 mg L^−1^, volume 20 mL, weight 0.03 g, contact time 30 min).

The maximum adsorption capacity of several obtained MNPs was studied by isotherms of adsorption (Fig. 13). The adsorption isotherms of Cu(ii), Cd(ii), Fe(iii) and Zn(ii) ions on MNPs are not similar to each other. At the low concentrations, the slopes are high and smoothing with increasing equilibrium concentration. According to the Giles classification, the isotherms can be classified as L1 type,^46^ indicating affinity of the adsorbent to the adsorbate. Thus, it can be concluded that chemisorption of the metal ions on MNPs occurs due to complexation with compounds covering the magnetic core.

According to the data (Fig. 13), the adsorption capacities of the Fe 4-1 (a) and Fe 5-1 (b) samples were about 29.1 mg g^−1^ (31.5 mg g^−1^) for Cd(ii), 25.5 mg g^−1^ (17.5 mg g^−1^) for Cu(ii), 24.0 mg g ^−1^ (13.9 mg g^−1^) for Zn(ii), and 13.1 mg g^−1^ for Fe(iii), respectively. The high adsorption capacity suggests that the high amount of active sites of the magnetic adsorbent was located on the external surface. The maximum adsorption capacity of Fe 4-1 for Cd(ii) was higher than that of Fe 5-1. This could be attributed to the greater amount of binding sites of Fe 4-1 for Cd(ii) compared to that of Fe 5-1. The observed differences in the capacities are most likely owing to their different affinities to interact with the surface of MNPs, according to the stability constant of complexes for each of the metal ions.

#### Selectivity and reusability

**Fig. 14:**
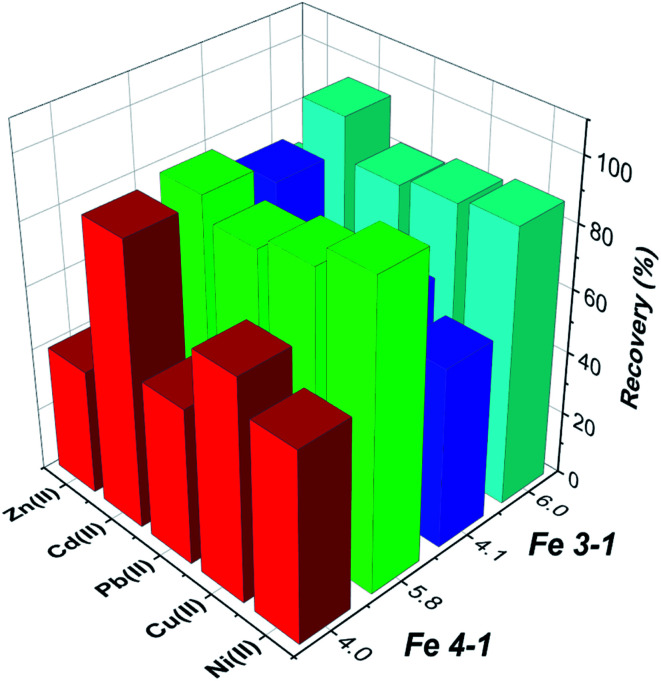
Competitive adsorption of selective metal ions from multi-element mixture on MNPs (conditions: pH 4.0 or 6.0, volume, 25 mL; initial concentration for each metal ion 20 mg L^−1^).

The selectivity and effects of potentially interfering ions on the removal efficiency of the obtained MNPs and subsequent AAS determination of metal ions were investigated. Multi-element standard solution for 23 elements was processed according to the bench adsorption experiments (Fig. 14). The tolerance of potentially interfering ions was defined as the amount with recovery of the studied elements at less than 85%. It was found that the studied metal ions could not be recovered quantitatively in the presence of Ca(ii), Fe(iii), Cr(iii), Sr(ii), Mg(ii) and Co(ii) ions.

The results shows that the presence of K, B, Ba, Bi, Mn, Na, and others have no obvious influence on the determination of the selected metal ions under the studied conditions, which shows that the tested MNPs have satisfactory selectivity for target metal ions.

Equally, it was experimentally proved that the complete desorption of metals is observed under treatment of a metal-containing adsorbent with 0.1 mol L^−1^ HNO_3_.

### 3.9 Application of the obtained MNPs for environmental remediation

The MNPs (Fe 4-1 and Fe 5-1 samples) have been used for the removal of Cu(ii), Co(ii), Ni(ii) and Cd(ii) ions from real river water. Environmental water samples were directly analyzed after acid conservation and filtration. The results for the spiked samples analysis are presented in Table 3.

**Table 3:** The results of the Zn(ii), Cu(ii), Ni(ii) and Cd(ii) determination in water samples after their removal on obtained MNPs, from river waters: Dnipro, Kiev (Ukraine). (Volume 200 mL, pH 6.5, weight 30 mg, desorption by HNO_3_ at pH 1.0, volume of eluate 2.0 mL)

Target ions	Fe 4-1 sample			Fe 5-1 sample		
	Spiked, μg L^−1^	Found, μg L^−1^	Recovery, %	Spiked, μg L^−1^	Found, μg L^−1^	Recovery, %
Zn(ii)	0	8.0 ± 0.4	—	0	7.5 ± 0.3	—
	6.6	15.1 ± 0.5	106	4.3	11.5 ± 0.1	95
Cu(ii)	0	10.5 ± 0.1	—	0	10.8 ± 0.2	—
	6.8	16.9 ± 0.5	91	2.0	12.7 ± 0.3	86
Ni(ii)	0	11.3 ± 0.5	—	0	11.3 ± 0.4	—
	7.3	18.9 ± 0.5	90	7.8	19.1 ± 0.2	103
Cd(ii)	0	10.1 ± 0.1	—	0	11.1 ± 0.2	—
	5.1	15.0 ± 0.1	96	2.0	13.0 ± 0.3	100

As can be seen (Table 3), the concentrations of Cd in the Dnipro river water were below the maximum residual level (MRL) (0.003 mg L^−1^), according to the water quality for drinking: WHO guidelines (2012). The results of the environmental water analysis were confirmed by spiked method and demonstrated good agreement between them. The experimental recovery of the studied metals was in the frame of 86–106% and slightly decreased (up to 86%) for low-contaminated water. The enrichment factor of metal ions in the real water samples was no less than 172. These results demonstrated the suitability the biosynthesized MNPs for the determination of trace metal ions in the environmental water samples.

## 4. Conclusions

In this study, an efficient and environmentally-friendly method of plant-based biosynthesis of Fe_3_O_4_-NPs and CoFe_2_O_4_-NPs using “hairy” roots of *Artemisia annua* L was proposed. The ability of ethanol extracts of wormwood “hairy” roots to ‘green’ synthesize MNPs from a mixture of salts was evaluated. It has been demonstrated that extracts of roots contain plant-synthesized components, which can transfer Fe(ii,iii) and Co(ii) ions into magnetite and cobalt ferrite nanoparticles with magnetic properties. The obtained Fe_3_O_4_-NPs solids exhibited the superparamagnetic properties. TEM images of the produced MNPs revealed their spherical and/or square shape with predominant sizes from 5 to 30 nm. XRD patterns confirmed that the MNPs were Fe_3_O_4_ (JCPDS no. 98-015-9975) with a face-centred cubic spinel crystalline structure. These results describe the first successful report on using “hairy” root extracts for the biosynthesis of Fe_3_O_4_ and CoFe_2_O_4_-NPs. The resulting MNPs demonstrated the significant inhibition effect on the roots growth. The synthesized MNPs effectively removed model metal ions from aqueous medium with digestible adsorption capacity, making them useful for environmental remediation.

## Conflicts of interest

There are no conflicts to declare.

## Supplementary Material

RA-011-D1RA04080D-s001
