# Supplementary material for: Biosynthesis of magnetite and cobalt ferrite nanoparticles using extracts of “hairy” roots: preparation, characterization, estimation for environmental remediation and biological application
Source: RSC Adv. 2021 Aug 9;11(43):26974–87. doi: 10.1039/d1ra04080d (PMC9037682; doi:10.1039/d1ra04080d)
Supplement: RA-011-D1RA04080D-s001 [file RA-011-D1RA04080D-s001.pdf]

Electronic Supplementary Material (ESI) for RSC Advances.
This journal is © The Royal Society of Chemistry 2021

Electronic Supporting Information

Biosynthesis of Magnetite and Cobalt Ferrite Nanoparticles using Extracts of 'Hairy' Roots: Preparation, Characterization, Estimation for Environmental Remediation and Biological Application

Natalia Kobylinska[1], Dmytro Klymchuk[3], Anatolij Shakhovsky[2], Olena Khainakova[4], Yakiv Ratushnyak[2], Volodymyr Duplij[2], Nadiia Matvieieva[2]

[1]A.V. Dumansky Institute of Colloid Chemistry and Water Chemistry NAS of Ukraine, Ak. Vernadsky blv. 42, Kyiv, 03142, Ukraine
[2]Institute of Cell Biology and Genetic Engineering, NAS of Ukraine, 148 Zabolotnogo Str, Kyiv, 03143, Ukraine
[3]M.G.Kholodny Institute of Botany, NAS of Ukraine, 2 Tereshchenkivska Str, Kyiv, 02000, Ukraine
[4]University of Oviedo, 8 Julián Claveria Av., Oviedo, 33006, Spain

Corresponding author: kobilinskaya@univ.kiev.ua

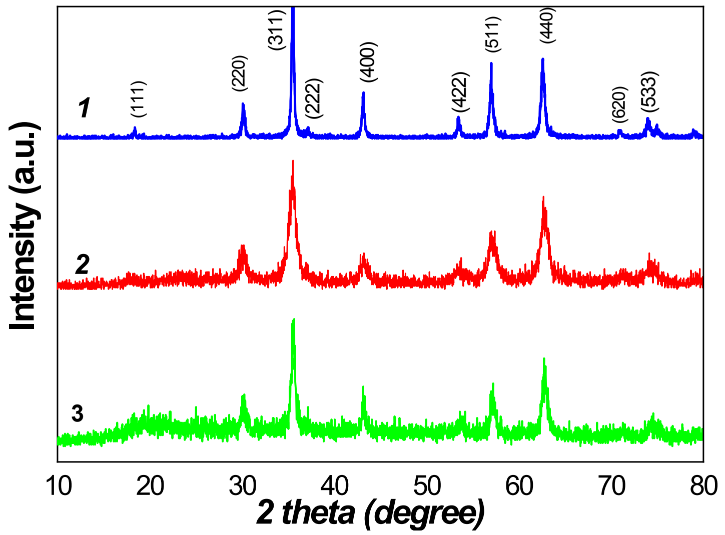

Fig. S1. Powder XRD patterns of Fe_3O_4-NPs (*Fe 4-1*) samples: 1 - washing by water and dried in vacuum; c - washing by water and dried in air at 25 °C; *d* - dried in air at 25 °C.

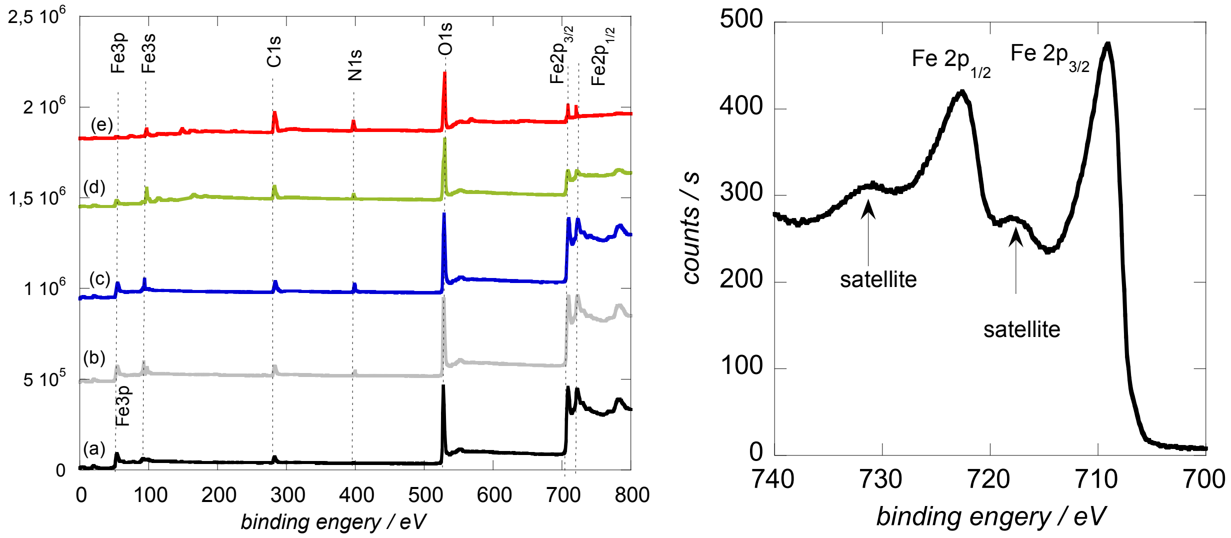

Fig. S2. XPS survey spectra *(a)* of as-prepared (wash-free) MNPs (a - *Fe 1-1,b - Fe 2-1, c - Fe 3-1,d - Fe 4-1* and *e - Fe 6-1* samples) and high resolution Fe2p spectrum *(b)* of as-synthesized MNPs (sample *Fe 4-1*).

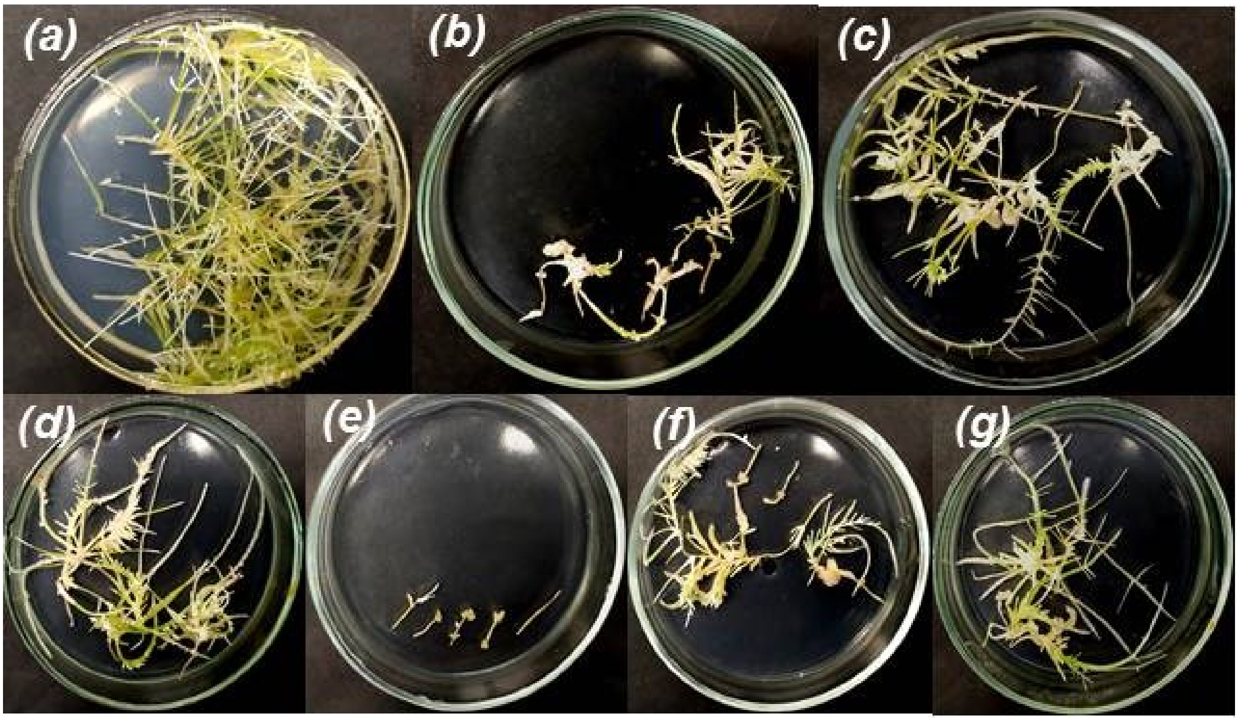

Fig. S3. Effect of MNPs to inhibit growth of *Althaea officinalis* ‘hairy’ roots: *a* – control without MNPs; *b* – Fe 1-1; *c* – Fe 2-1; *d* - Fe 3-1; *e* - Fe 4-1; *f*- Fe 5-1; *g* - Fe 6-1.
